# 20(*S*)-Protopanaxadiol Saponins Mainly Contribute to the Anti-Atherogenic Effects of *Panax notoginseng* in ApoE Deficient Mice

**DOI:** 10.3390/molecules24203723

**Published:** 2019-10-16

**Authors:** Conghui Liu, Ruibing Feng, Jian Zou, Fangbo Xia, Jian-Bo Wan

**Affiliations:** State Key Laboratory of Quality Research in Chinese Medicine, Institute of Chinese Medical Sciences, University of Macau, Taipa, Macao 999078, China; mb75804@um.edu.mo (C.L.); yb57513@um.edu.mo (R.F.); yb87505@um.edu.mo (J.Z.); yb67523@um.edu.mo (F.X.)

**Keywords:** *Panax notoginseng*, 20(*S*)-protopanaxadiol saponins, 20(*S*)-protopanaxatriol saponins, atherosclerosis, vascular inflammation

## Abstract

Atherosclerosis mainly contributes to cardiovascular disease, a leading cause of global morbidity and mortality. *Panax notoginseng* saponins (PNS) are proved to therapeutically attenuate the formation of atherosclerotic lesions. According to different sapogenin, PNS are generally classified into 20(*S*)-protopanaxadiol saponins (PDS) and 20(*S*)-protopanaxatriol saponins (PTS). It was reported that PDS and PTS might exert diverse or even antagonistic bioactivities. In this study, the probable effects of PTS and PDS on atherosclerotic development were investigated and compared in ApoE-deficient mice (ApoE^−/−^). Male mice were gavaged daily by PNS (200 mg/kg/d), PTS (100 mg/kg/d), or PDS (100 mg/kg/d), respectively for eight weeks. The treatments of PNS and PDS, but not PTS, showed decreased atherosclerotic lesions in the entire aorta by 45.6% and 41.3%, respectively, as evaluated by an en-face method. Both PNS and PDS can improve the plaque vulnerability, as evidenced by the increased collagen fiber, increased expression of α- smooth muscle actin (α-SMA), and decreased Cluster of differentiation 14 (CD14). Additionally, PDS also inhibit the nuclear factor kappa B (NF-κB)-mediated vascular inflammation in the aorta. In conclusion, PDS, but not PTS, might mainly contribute to the anti-atherosclerosis of *P. notoginseng*.

Academic Editor: Stefano Dall’Acqua

## 1. Introduction

Cardiovascular diseases (CVD) are the leading cause of morbidity and mortality globally. About 17.9 million deaths, representing 31% of all deaths worldwide, were attributed to CVDs in 2016 [1]. Atherosclerosis (AS), a multi-factorial disease, is the most common contributor to CVD [2]. Increasing evidence has indicated that chronic vascular inflammation is closely implicated in the initiation and progression of atherosclerotic plaques [3,4]. Despite statins, the 3-hydroxy-3-methylglutaryl coenzyme A (HMG-CoA) reductase inhibitors with lipid-lowering capability are recommended for the prevention and treatment of AS, the overdose or long-term statin use may increase the risk of serious side effects, such as hepatotoxicity and rhabdomyolysis [5]. Thus, it is essential to develop the anti-atherogenic agents with multi-targets and less adverse effect from natural products or herbal medicines.

The roots of *Panax notoginseng* (Burk.) F. H. Chen is a highly valued Chinese medicine and has been clinically prescribed to treat CVD in Asian countries, either alone or in combination. *Xueshuantong* and *Xuesaitong* injections, made from total saponins isolated from the roots and rhizomes of *P. notoginseng*, respectively, are the creditable medicines to treat angina pectoris, coronary heart disease, and arrhythmia cordis [6]. Dammarane triterpene saponins (refer to ginsenosides) are identified as the main bioactive ingredients behind the claims of *P. notoginseng*. A large body of studies has indicated that *P. notoginseng* saponins (PNS) and its individual ginsenosides efficiently attenuate the atherosclerotic lesion in apolipoprotein E-deficient (ApoE^−/−^) mice [3,7,8,9,10] and zymosan A-induced atherosclerotic mice [11,12,13,14]. Based on the different structural skeletons of sapogenin, ginsenosides identified in *P. notoginseng* are generally classified into two subgroups: 20 (*S*)-protopanaxadiol saponins (PDS, including ginsenosides Rb1 and Rd) and 20(*S*)-protopanaxatriol saponins (PTS, including notoginsenoside R1, ginsenosides Rg1 and Re). Interestingly, it was reported that PDS and PTS might exert diverse or even antagonistic bioactivities [15,16,17]. Our previous study indicated that PDS exhibit more potent effects on the inhibition of vascular inflammation induced by tumor necrosis factor α (TNF-α) *in vitro*, suggesting the better efficacy in anti-atherosclerosis, compared to the PTS [18]. However, their anti-atherosclerotic effects in vivo and the mechanisms behind them are largely unknown. Therefore, the aim of the present study is to investigate and compare the probable effects of PDS and PTS on atherosclerotic development in ApoE^−/−^ mice.

## 2. Results

### 2.1. Chemical Characteristics of the Tested Samples

**Figure 1:**
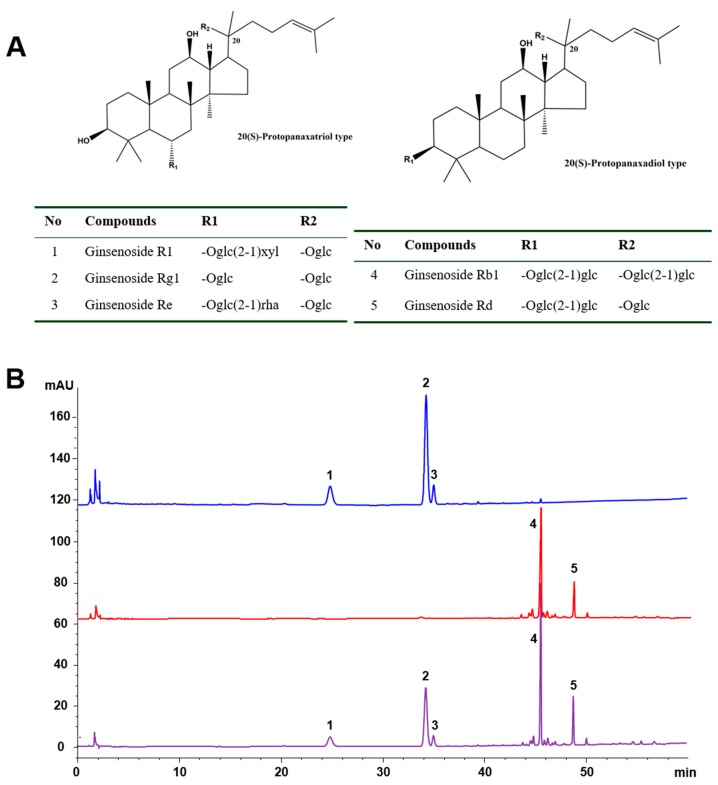
(**A**) Chemical structures of the main saponins in *P. notoginseng*, and (**B**) the HPLC-UV profiles of 20(*S*)-protopanaxatriol saponins (PTS), 20(*S*)-protopanaxadiol saponins (PDS), and *Panax notoginseng* saponins (PNS). 1, notoginsenoside R1; 2–5, ginsenosides Rg1, Re, Rb1, and Rd, respectively.

PDS and PTS were previously prepared from commercial PNS using microporous absorptive resins in our lab [19]. To ensure the reproducibility of efficacy, the chemical profiles of the saponin fractions tested, including PNS, PDS, and PTS, were determined by the high-performance liquid chromatography (HPLC) method as described [20]. Five saponins, named notoginsenoside R1, ginsenosides Rg1, Re, Rb1, and Rd (Figure 1A), were identified to be the main ingredients in PNS, their total contents constituted 90.4% of PNS. The former three saponins (i.e., R1, Rg1, and Re), and the latter two saponins (i.e., Rb1 and Rd) were the main components of PTS and PDS, respectively. Furthermore, these saponins constituted 87.4% and 91.8% of PTS and PDS fractions, respectively (Figure 1B).

### 2.2. Effects of PNS, PDS, and PTS on Atherosclerotic Lesions

**Figure 2:**
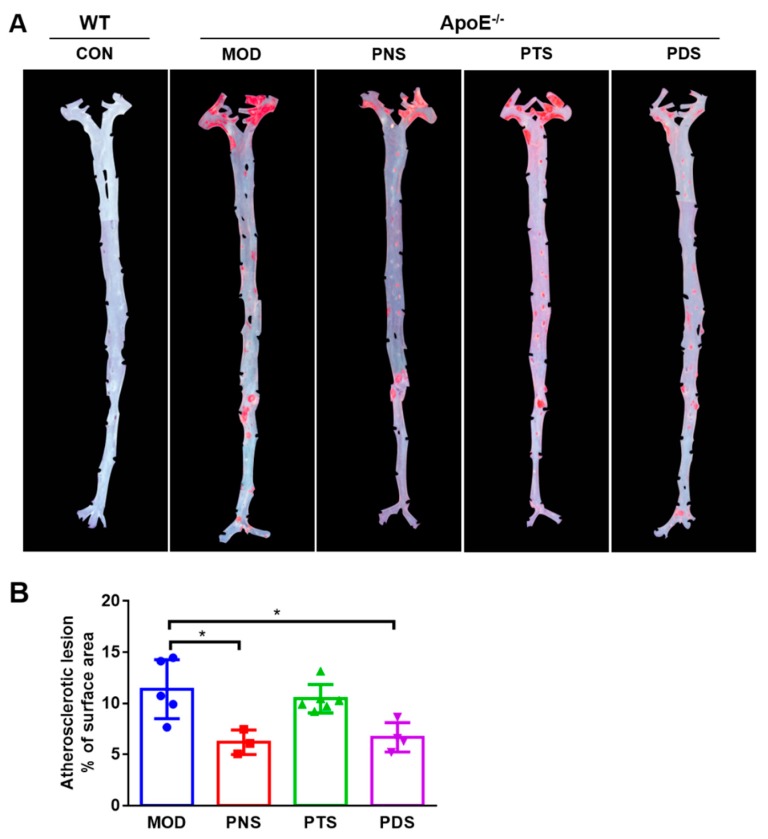
Atherosclerotic lesions in the entire aorta from wild-type mice (CON), untreated ApoE^−/−^ mice (MOD), and PNS/PTS/PDS-treated ApoE^−/−^ mice. (**A**) Representative Oil red O-stained, longitudinally opened aorta, atherosclerotic plaques (red). (**B**) The percentage of the lesion area of the entire aorta. n = 4–6 per group. *, *p* < 0.05.

**Figure 3:**
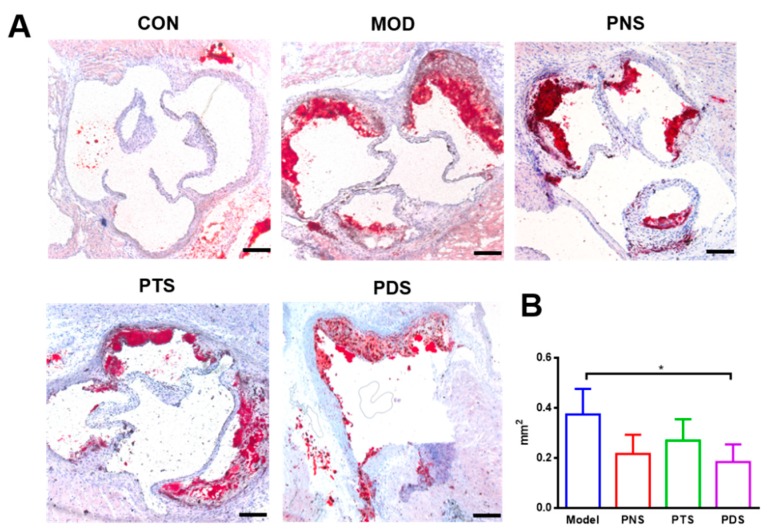
(**A**) Representative cross sections of the aortic sinus from all groups stained by Oil red O, and (**B**) the histogram of calculated lesion sizes (n = 3–4). Scale bar, 200 μm. *, *p* < 0.05.

After 12 weeks of feeding with a western-type diet, severe atherosclerotic plaques were clearly observed in the entire aorta from ApoE^−/−^ mice, as measured by the en-face method. Oil red O staining of the entire aortas indicated that the prominent lipid-rich lesions (red) in PNS and PDS groups, but not PTS, were obviously reduced, compared with the model group (MOD), which was confirmed by the quantitative analysis (Figure 2B). The percentage of the Oil red O-stained lesion area to entire aorta area in the mice from PNS (6.19 ± 1.21%) and PDS (6.68 ± 1.43%) groups were remarkably lower than those from the MOD group (11.38 ± 2.89%) by 45.6% and 41.3%, respectively. However, there was no significant difference in atherosclerotic lesions between the PTS (10.45 ± 1.38%) and MOD groups. Similar results were observed by the cross-sectional histological analysis. The atherosclerotic plaques stained with Oil red O in the aortic sinus were less prominent in the PNS and PDS groups over the MOD group (Figure 3A). The quantitative analysis (Figure 3B) was shown that the average positive areas stained by Oil red O in the PNS, PTS, and PDS groups were less than that in the MOD group by 50.84%, 27.85%, and 42.10%, respectively, but the significant difference was only observed between the PDS and MOD groups.

### 2.3. Levels of Plasma Lipids

To examine whether the protective effects of PNS and PDS are attributed to their lipid-lowering properties, the plasma lipid profiles were determined by their commercial kits (Table S2). Compared to the wild-type mice as the control group (CON), the MOD group exhibited significant increases in plasma levels of total triglyceride (TC), total cholesterol (TG), and low-density lipoprotein (LDL), and a decrease in plasma high-density lipoprotein (HDL) levels. However, the treatments of PNS and PTS at the given dosage appears not to change the plasma lipid parameters in ApoE^-/-^ mice. PDS-treated group decreased the plasma levels of TG and LDL, but not significantly, compared to the MOD group.

### 2.4. Effects of PNS, PDS, and PTS on Plaque Vulnerability

**Figure 4:**
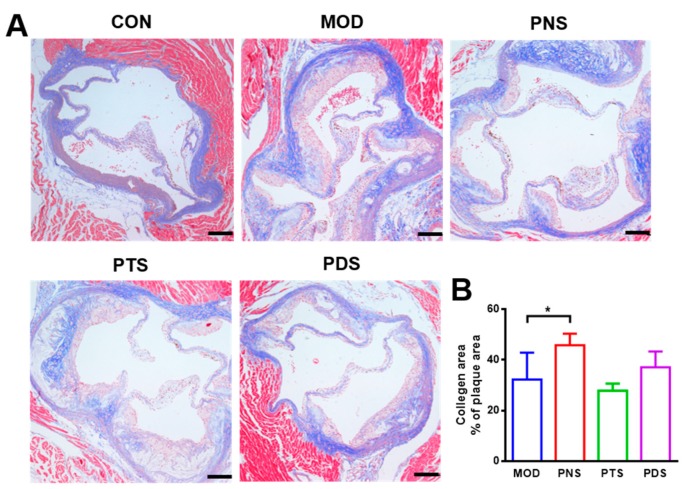
Representative of Masson’s trichrome staining sections (**A**) and the quantification of collagen areas to plaque areas (**B**). Scale bar, 200 μm (n = 5–7). *, *p* < 0.05.

**Figure 5:**
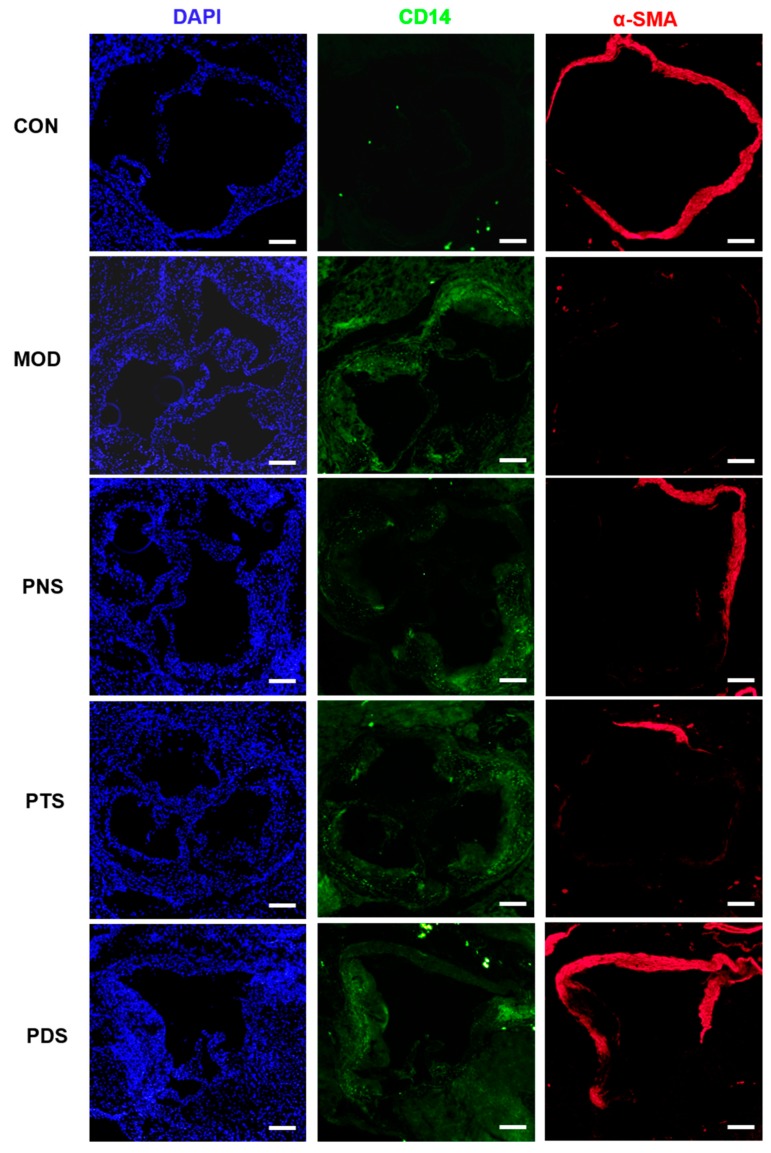
Representative immunofluorescent staining of DAPI (blue), CD14 (green), and α-SMA (red) in the cross section of the aortic sinus. Scale bar, 200 μm.

In order to investigate the effects of saponin fractions on plaque vulnerability, collagen fibers in atherosclerotic plaque were assessed by Masson’s Trichrome staining. Cluster of differentiation 14 (CD14), the marker of macrophage, and α-smooth muscle actin (α-SMA), the marker of smooth muscle cells (SMCs) on the atherosclerotic plaques, were examined by immunofluorescent staining, respectively. As shown in Figure 4, the treatment of PNS can significantly increase the component ratio of the collagen area to plaque area (45.81 ± 4.54%), compared with the MOD group (32.3 ± 10.57%). Although no significant difference was observed between PDS and MOD, the average content of the collagen area in the lesions from the PDS group was higher than that from the MOD group, and PTS did not show any effect. Additionally, a more severe CD14-positive macrophage was observed in atherosclerotic lesions from the MOD group compared to the CON group. This macrophage infiltration was obviously reduced by the treatment of either PNS or PDS, but not PTS (Figure 5). α-SMA, the SMC marker, indicates the integrity of the arterial wall and fibrous cap. The stronger α-SMA staining in the plaque from PNS and PDS-treated mice was observed, indicating less impaired integrity of the arterial wall over the mice in the MOD group. The PTS-treated group showed a slightly alleviative effect.

### 2.5. Effects of PNS, PDS, and PTS on Vascular Inflammation

**Figure 6:**
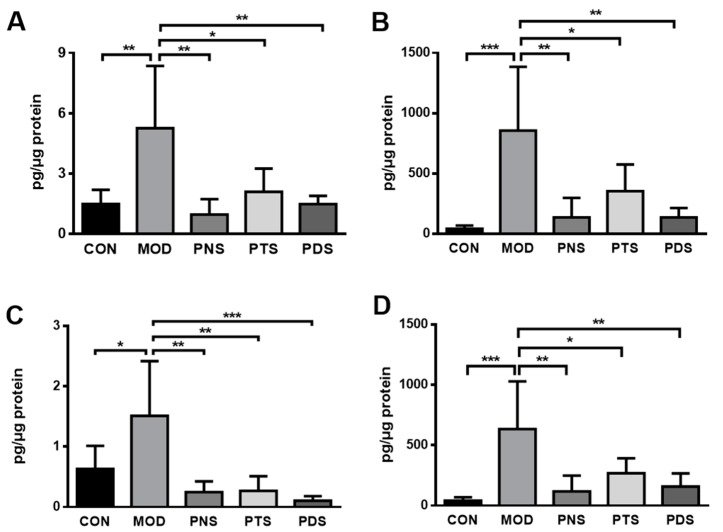
The productions of TNF-α (**A**), IL-6 (**B**), IL-1β (**C**), and MCP-1 (**D**) in the ex vivo culture medium of the aortic arch. Data were presented as mean ± SD (n = 6). *, *p* < 0.05; **, *p* < 0.01; ***, *p* < 0.001.

Vascular inflammation plays a pivotal role in the initiation and progression of atherosclerosis. To investigate whether the protective effects of PNS and PDS are associated with vascular inflammation, the secretion of cytokines in the aorta culture medium were measured using *ex vivo* tissue culture. To normalize differences in tissue size, we extracted and quantified the protein of tissues after the incubation for 24 h. As shown in Figure 6, the western-type diet caused severe vascular inflammation in the aorta from ApoE^−/−^ mice, as evidenced by the dramatically increased levels of pro-inflammatory cytokines, such as TNF-α, interleukin 6 (IL-6) and IL-1β, and monocyte chemoattractant protein-1 (MCP-1), in the culture medium of the isolated aorta. These elevations were significantly reduced by the treatments of PNS, PTS, or PDS.

**Figure 7:**
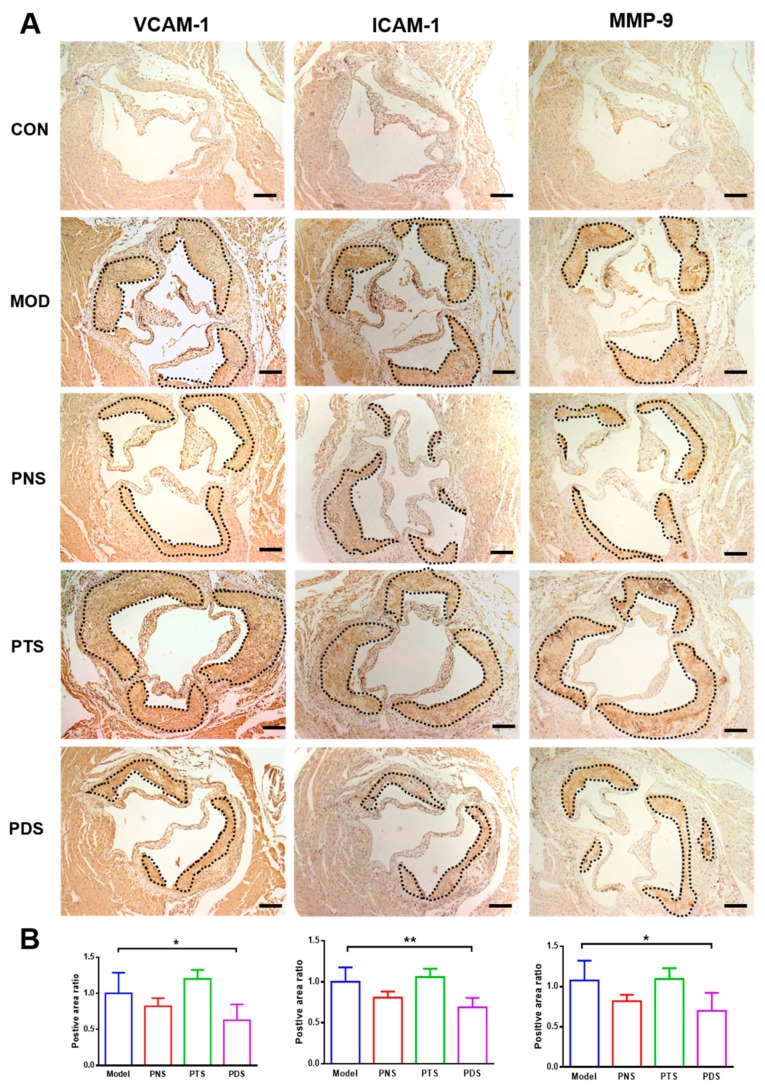
Immunohistochemical staining of VCAM-1, ICAM-1, and MMP-9 in cross sections of the aortic sinus (**A**) and corresponding quantifications (**B**). The stained area was cycled by black dash lines. Scale bar, 200 μm. Data were presented as mean ± SD (n = 5–7). *, *p* < 0.05; **, *p* < 0.01.

Cellular adhesion molecules (CAMs), such as intercellular adhesion molecule 1 (ICAM-1) and vascular cell adhesion molecule 1 (VCAM-1), contribute to the recruitment and migration of circulating monocytes into the vessel wall, leading to a vascular inflammatory response in atherosclerosis. Next, the protein expressions of ICAM-1, VCAM-1, and matrix metallopeptidase 9 (MMP-9) were also measured by immunohistochemistry analysis. As shown in Figure 7, the protein expressions of ICAM-1, VCAM-1, and MMP-9 were greatly upregulated in the plaque from untreated ApoE^−/−^ mice, and these up-regulations were significantly inhibited by the treatment of PDS, but not PTS. Although the PNS-treated group showed a decreasing tendency, it was not significantly different from the MOD group.

**Figure 8:**
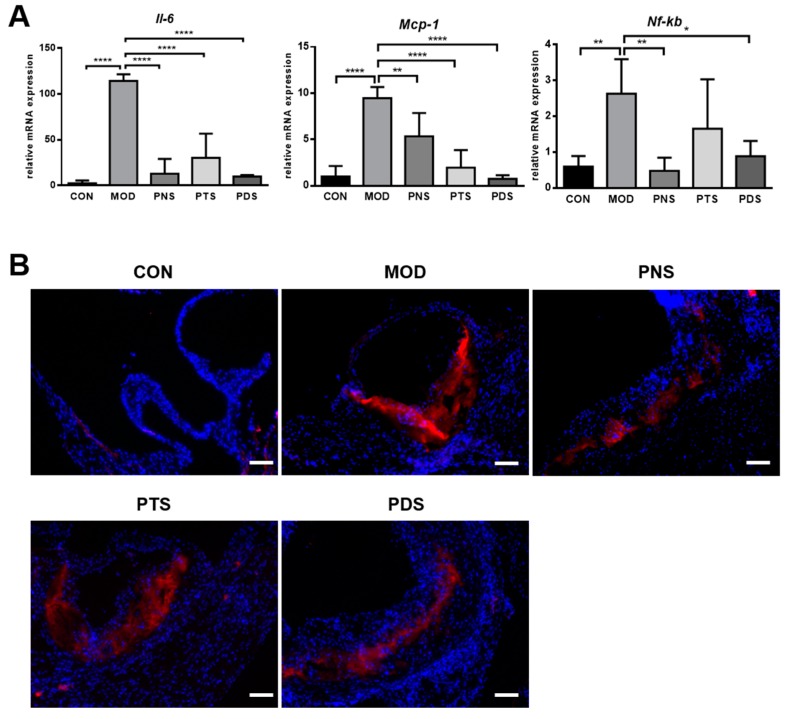
The transcriptional expression of *Il-6, Mcp-1*, and *Nf-κb* in the entire aorta tissue (**A**), and the representative immunofluorescent staining of p65 (red) in the frozen section of the aortic sinus (**B**). Data in qPCR were normalized to the CON group (n = 5–6). *, *p* < 0.05; **, *p* < 0.01; ****, *p* < 0.0001. Scale bar, 100 μm; nuclei were stained with DAPI (blue).

Additionally, to examine whether the saponin fraction affects the expression of inflammation-related genes, the transcriptional expressions of several key factors in the aorta were examined by qPCR. As shown in Figure 8A, the mRNA expressions of IL-6, MCP-1, and nuclear factor kappa B (NF-κB) was greatly up-regulated in the aorta from un-treated ApoE^−/−^ mice compared with CON mice, and the increased expressions of all these genes tested were inhibited by the treatment of PNS, PTS, or PDS, although the changes in NF-κB expression did not reach statistical significance between the MOD and PDS groups. NF-κB is a key transcriptional factor for regulating the expressions of several inflammation-related genes in atherosclerosis, including chemokines, cytokines, and CAM [21]. We subsequently examined the protein expression of p65, the most abundant component of the NF-κB family, in the aorta by immunofluorescent staining. The expression of p65 in the aorta from ApoE^−/−^ mice was obviously reduced by the treatment of PNS or PDS, but not PTS.

## 3. Discussion

Ginsenosides are widely considered as bioactive ingredients responsible for the anti-atherosclerotic effects of *P. notoginseng*. According to their aglycones, ginsenosides isolated from *P. notoginseng* are generally classified into PDS and PTS. An increasing number of studies have demonstrated that PDS and PTS may exert different or even antagonistic bioactivities [15,16,17,22]. PDS in the *P. ginseng* have shown hemolytic activity, while PTS possess an anti-hemolytic effect. Consequently, the total saponins composed of PDS and PTS have no or very mild hemolytic properties. Ginsenosides Re and Rg1, belonging to PTS, have the ability to promote angiogenesis, while, ginsenosides Rb1, Rg3, and Rh2, belonging to PDS, shown anti-angiogenesis effects [23,24,25]. Ginsenoside Rb1, but not Rg1, suppressed abdominal aortic aneurysm (AAA) in Ang II-infused ApoE^−/−^ mice through inhibiting JNK and p38 signaling pathways [22]. Our previous study has also shown that PDS exert more potent protective effects than PTS on TNF-α-induced vascular inflammation in human umbilical vein endothelial cells (HUVECs) [18]. However, the anti-atherosclerotic effects of PDS and PTS have not been addressed yet. Therefore, in the present study, we demonstrated that oral administration of PDS, but not PTS, notably decreased the formation of atherosclerotic plaques in ApoE^−/−^ mice fed a western-type diet, as evidenced by both the en-face method and cross-section staining. The protective effects of PDS are attributed to the enhanced plaque stability and reduced vascular inflammation in mice, independent of a lipid-lowering effect. These findings suggest that PDS might be the main bioactive ingredients responsible for the anti-atherosclerotic effect of *P. notoginseng.*

Hyperlipidemia is a prevalent risk factor of atherosclerotic CVD. After feeding a western diet for 12 weeks, the ApoE^–/–^ mice in the MOD group developed severe hyperlipidemia, as indicated by higher plasma levels of TC, TG, and LDL, as well as lower HDL, compared to CON mice. In the present study, no significant differences in plasma lipid parameters were observed between MOD and the three treatment groups (Table S2). These observations are consistent with the results in previous studies [8,26,27], but disagreed with several reports [3]. An explicative hypothesis proposed is that different doses of PNS and varied modeling were applied in our study. Genetic deficiency of ApoE in mice leads to the main defects in the clearance capability of TG and VLDL from blood circulation. These results suggest that PNS, PTS, or PDS at the given doses cannot efficiently improve the circulating blood lipid profile in ApoE^−/−^ mice. The mean values of plasma TG and LDL levels in the PDS group were lower than the MOD group, but there is no significant difference. Despite that the hypolipidemic effect has been considered as an anti-atherosclerotic mechanism, it seems that the anti-atherosclerotic effects of PNS and PDS observed in the present study are independent of the lipid-lowering effect.

Plaque vulnerability is an important risk factor in producing sudden major problems, such as stroke or heart attack. Collagen fibers, the primary component of atherosclerotic plaque, have been widely used as an indicator to evaluate plaque stability [28]. The morphometric ‘vulnerability index’ was also proposed by Shiomi et al. [29], which involved macrophage infiltration, collagen fibers, and SMCs in the atherosclerotic plaque [30]. In this study, the treatment of PNS and PDS, but not PTS, increased the collagen fiber ratio in atherosclerotic plaque and the expression of α-SMA, the marker of SMC, and decreased the expression of CD14, the marker of macrophages on the atherosclerotic plaques. These data show that PNS and PDS can enhance plaque stability.

It has been well documented that vascular inflammation in the arterial wall plays a vital role in the pathogenesis of atherosclerosis [3]. Vascular inflammation is characterized by the infiltration of macrophages and the increased production of cytokines. Atherogenic lipoproteins, such as LDL and oxidized LDL, activate vascular endothelial cells, resulting in the up-regulation of endothelial adhesion molecules and initiation of vascular inflammation. Subsequently, the circulating monocytes are activated and recruited into the artery wall and differentiate to macrophages, accelerating atherosclerotic lesion growth and exacerbating the vascular inflammatory response. These inflammatory factors, such as cytokines, chemokines, adhesion molecules, and macrophage infiltration, are well controlled by NF-κB in atherosclerosis. Our present study clearly demonstrates that PNS, PTS, and PDS have inhibitory effects on vascular inflammation, as evidenced by less macrophage infiltration, the decreased production of TNF-α, IL-6 and IL-1β, and MCP-1 in culture medium from the isolated aorta, and reduced expression of inflammation-related genes in the aorta, including IL-6, MCP-1, and NF-κB. However, the protein expressions of ICAM-1, VCAM-1, and MMP-9 up-regulated by western-diet feeding were significantly inhibited by the treatment of PDS, but not PTS. Although the PNS-treated group showed a decreasing tendency, it was not significantly different from the MOD group. Our results clearly demonstrated that PDS, but not PTS, showed decreased atherosclerotic lesions in the entire aorta, as evaluated by both en-face and cross-section methods. However, our data also suggest that both PDS and PTS exhibit vascular anti-inflammation significantly. The potential mechanisms underlying the different effects of PDS and PTS remains to be further elucidated. Ginsenoside Rb1 and Rd, belonging to PDS, were shown to have anti-atherosclerotic effects by anti-Ca2^+^ entry and ROS clearance [10,31], and enhancement of macrophage autophagy [32]. Furthermore, notoginsenoside R1, belonging to PTS, was also shown to attenuate the atherosclerotic lesions in apoE^−/−^ mice via the inhibition of inflammation and oxidative stress [33]. To our best knowledge, this is the first study to elucidate and compare the anti-atherosclerotic effects of different types of ginsenosides from *P. notoginseng*.

Taken together, our study demonstrated that PDS, but not PTS, reduced the formation of atherosclerotic plaques in ApoE^−/−^ mice, likely because of the enhanced plaque stability and anti-inflammatory effects of PDS. Our findings suggest that PDS might mainly contribute to the anti-atherosclerosis of *P. notoginseng.* The anti-atherogenic effect of PDS remains to be further validated by other animal models and clinical trials, and the potential mechanisms underlying the different effects of PDS and PTS need to be further investigated.

## 4. Materials and Methods

### 4.1. HPLC-UV Analysis of PNS, PTS, and PDS

PNS was purchased from Wanfang Natural Pharmaceutical Co. (Yuxi, Yunnan, China). PDS and PTS were previously separated from PNS using DS-401 macroporous resins, as established by our lab [19]. All tested samples, including PNS, PDS, and PTS, were analyzed by HPLC-UV, as described previously [20]. An Agilent 1100 liquid chromatography system (Palo Alto, CA, USA) was applied for HPLC analysis, with a Zorbax ODS C_18_ column (4.6 mm × 250 mm, 5 μm) and a guard column (4.6 mm × 12.5 mm, 5 μm). A binary gradient elution system consisting of water (A) and acetonitrile (B) was performed with the following gradient program: 0–30 min, 18%–19% B; 30–35 min, 19%–35% B; 35–60 min, 35%–55% B. The flow rate and column temperature were 1.5 mL/min and 40 °C, respectively. The detection wavelength was set at 203 nm. Acetonitrile (HPLC-grade) was provided from Fisher Scientific (Pittsburgh, PA, USA), and the water was purified with a Milli-Q water purification system (Millipore, Bedford, MA, USA). The reference standards, including notoginsenoside R1, ginsenosides Rg, Re, Rb1, and Rd, were previously prepared by our laboratory (Figure 1A) [34].

### 4.2. Animals and Treatment

Homozygous mice with the Apoe^tm1Unc^ mutation with a C57BL/6J genetic background (B6.129p2-Apoe ^tm1Unc^/J) were purchased from the Jackson Laboratory (Bar Harbor, ME, USA). ApoE^−/−^ and C57BL/6J mice were housed in a specific pathogen-free animal facility, Faculty of Health Science, University of Macau. At the age of 6 weeks, the male mice were fed a western-type diet (consisting of 21% fat and 0.2% cholesterol, Trophic Animal Feed High-Tech Co., Ltd., Nantong, Jiangsu, China) for 12 weeks. In the last 8 weeks, the ApoE^−/−^ mice in treatment groups were gavaged daily by PNS (200 mg/kg/d), PTS (100 mg/kg/d), or PDS (100 mg/kg/d) aqueous solutions, respectively. Wild-type mice as CON and apoE^−/−^ mice in MOD received an equal volume of distilled water. At the endpoint, all animals were sacrificed with isoflurane (Abbott Laboratories, Chicago, IL, USA), and their blood samples were promptly collected by a cardiac puncture for lipid analysis. Animal research protocols (No. UMARE-020-2017) were approved by the Animal Research Ethics, University of Macau.

### 4.3. Measurement of Atherosclerotic Lesions

The atherosclerotic lesions in the entire aorta and aortic sinus were assessed by the en-face method and cross-section method, respectively [35]. After perfusion with cold phosphate-buffered saline solution, the entire aorta with main branches were immediately dissected from the heart up to 3–5 mm from the iliac bifurcation under a dissecting microscope. The connective tissue and fat pad adhering to the aorta were removed as much as possible. After fixing with 4% paraformaldehyde solution overnight at 4 °C, the clean aorta was opened longitudinally and pinned onto a standard black wax dissection pan using microneedles (RWD Life Science Co., Ltd., Shenzhen, Guangdong, China). Subsequently, lipid-rich atherosclerotic lesions were stained with Oil O Red (Soliabio, Beijing, China). Aorta images were captured by an industrial Digital Camera (Nikon Corp., Tokyo, Japan) and analyzed by Image-Pro Plus software (v5.1, Media Cybernetics, Silver Spring, MD, USA). The ratio of the lesion area from the total aorta area in each mouse was calculated.

The cross-sectional plaque in the aortic sinus was also measured as described previously [35]. A fresh heart sample with the aortic sinus was embedded in an OCT matrix. The sample was sectioned serially (10 μm interval) by a Leica CM1950 cryostat (Leica Camera Inc. Allendale, NJ, USA). After staining by oil red O, the specimens were recorded with an Olympus IX73 microscope (Olympus Corp., Tokyo, Japan).

### 4.4. Plasma Lipid Analysis

All blood from the mice was collected using a heparin sodium-pretreated tube, and the plasma was separated by standard protocol. Plasma levels of TC, TG, LDL, and HDL were assayed by their corresponding kits, respectively (Nanjing Jiancheng Bioengineering Institute, Nanjing, Jiangsu, China) according to the manufacturer’s protocols.

### 4.5. Ex Vivo Aorta Tissue Culture and Cytokine Assays

The fresh ascending aortic arch segment from each group was dissected and cut into 2 mm-rings in 1 mL of serum-free DMEM, supplemented with penicillin (100 IU/mL) and streptomycin (100 μg/mL). The tissue was incubated for 24 h at 37 °C, then the culture supernatant and remaining aorta tissue were collected and stored at −80 °C before analysis. The concentrations of inflammatory cytokines, including TNF-α, IL-6, IL-1β, and MCP-1, in the culture medium were individually determined by their Mouse Enzyme-Linked Immunosorbent Assay MAX^TM^ Standard Kits (Biolegend Inc., San Diego, CA, USA). The total protein in the remaining aorta was measured by a Pierce™ BCA Protein Assay Kit (Fisher Scientific). Their cytokine levels were normalized to aorta total protein.

### 4.6. Immunostaining Assay

Immunofluorescent staining of CD14, α-SMA, and p65 was conducted as previously described [36]. The cryostat sections (10 μm) of the aorta sinus were fixed in 4% cold paraformaldehyde and washed with PBS (pH 7.4). After blocking, the slides were incubated with rabbit anti-mouse p65 (1:100, Cell Signaling Technology, Inc., Danvers, MA, USA), rabbit anti-mouse CD14 (1:100, abcam, Cambridge, MA, USA), and α-SMA (1:100, abcam, Cambridge, MA, USA) overnight at 4 °C. After washing by PBS, the sections were further incubated with Alexa Fluor^®^ 568 goat anti-rabbit IgG (1:200, abcam) or Alexa Fluor^®^ 488 anti-rabbit IgG (1:200, abcam, Cambridge, MA, USA) for 2 h at 37 °C in the dark. Subsequently, the cellular nuclei were counterstained with DAPI (1:1000, Sigma-Aldrich, Darmstadt, Germany).

Immunohistochemistry analysis of ICAM-1, VCAM-1, and MMP-9 were also conducted as described [36]. The paraffin sections were dewaxed and retrieved for antigen with gradient organic solvents and EDTA (pH 9.0), and then blocked with 3% H_2_O_2_ (aq) for 25 min at room temperature, the sections were incubated with anti-mouse ICAM-1, VCAM-1, and MMP-9 (1:100, Proteintech Group Inc., Wuhan, China). Staining was detected with an I-View DAB detection system (Servicebio, Beijing, China). After staining by hematoxylin and dehydrating, the stained specimens were recorded with an Olympus IX73 microscope and analyzed using ImageJ software (National Institutes of Health, Bethesda, MD, USA).

### 4.7. qPCR Assay

Total RNA from the entire aorta tissue was extracted by Trizol reagent according to the manufacture’s protocol (Invitrogen^TM^, Carlsbad, CA, USA). The purified RNA was reverse-transcribed to cDNA using the FastKing RT reagent kit with gDNA Eraser (Tiangen Biotech, Beijing, China). PCR reactions were performed by the SYBR Green^®^ qPCR kit (Tiangen Biotech, Beijing, China) on an Mx3005P qPCR system (Agilent Technologies, Palo Alto, CA, USA), and normalized to *Gapdh*. The sequences of the primers were shown in Table S1.

### 4.8. Statistical Analysis

All data were expressed as mean ± standard deviation (SD). Students’ t-test was conducted to test the difference between groups by GraphPad Prism software (v6.0, San Diego, CA, USA). Differences between groups were considered statistically significant at *p* < 0.05.

## Supplementary Materials

The following are available online at https://www.mdpi.com/1420-3049/24/20/3723/s1, Table S1: Primer sequences in qPCR; Table S2: Blood lipid profiles in the mice.
