# Supplementary material for: 20(S)-Protopanaxadiol Saponins Mainly Contribute to the Anti-Atherogenic Effects of Panax notoginseng in ApoE Deficient Mice
Source: Molecules. 2019 Oct 16;24(20):3723. doi: 10.3390/molecules24203723 (PMC6832312; doi:10.3390/molecules24203723)
Supplement: Supplementary file 1 [file molecules-24-03723-s001.pdf]

Supplementary Materials

20(*S*)-protopanaxadiol saponins mainly contribute to the antiatherogenic effects of *Panax notoginseng* in ApoE deficient mice

Conghui Liu, Ruibing Feng, Jian Zou, Fangbo Xia, Jian-Bo Wan*

State Key Laboratory of Quality Research in Chinese Medicine, Institute of Chinese Medical Sciences, University of Macau, Taipa, Macao, China

*Corresponding author:

Prof. Jian-Bo Wan,

Email: jbwan@um.edu.mo

Institute of Chinese Medical Sciences,

University of Macau,

Avenida da Universidade, Taipa, Macao, P.R. China

Supplementary Tables

Supplementary Table S1 Primer sequences in qPCR

Genes	Primer Sequences (5′→3′)	Size
MCP-1	Forward: TTAAAAACCTGGATCGGAACCAA	121
	Reverse: GCATTAGCTTCAGATTTACGGGT	
IL-6	Forward: TAGTCCTTCCTACCCCAATTTCC	133
	Reverse: TTGGTCCTTAGCCACTCCTTC	
NF-κB	Forward: TGGCCGTGGAGTACGACAA	69
	Reverse: TGTCCACGTGGGCATCAC	
GAPDH	Forward: AGGTCGGTGTGAACGGATTTG	95
	Reverse: GGGGTCGTTGATGGCAACA	

Supplementary Table S2. Blood lipid profiles in the mice.

Group	TC	TG	LDL	HDL
CON	4.07±0.95	0.88±0.36	1.01±0.60	3.81±0.84
MOD	17.08±4.26****	2.53±0.71****	13.36±2.55****	0.71±0.28****
PNS	17.81±3.05	2.27±0.67	13.17±3.12	0.79±0.31
PTS	19.18±4.30	2.61±0.93	15.12±3.40	0.90±0.29
PDS	17.24±5.57	1.74±0.52	9.81±3.40	0.94±0.32

Data are expressed as means ± SD (n=10-13). ****, $p<0.0001$ versus CON group
